# Self-prioritization during stimulus processing is not obligatory

**DOI:** 10.1007/s00426-019-01283-2

**Published:** 2020-01-09

**Authors:** Siobhan Caughey, Johanna K. Falbén, Dimitra Tsamadi, Linn M. Persson, Marius Golubickis, C. Neil Macrae

**Affiliations:** 1 School of Psychology, University of Aberdeen, Aberdeen, AB24 3FX Scotland, UK; 2 Department of Psychology, University of Toronto, Toronto, Canada

## Abstract

An emerging literature has suggested that self-relevance automatically enhances stimulus processing (i.e., the self-prioritization effect). Specifically, during shape–label matching tasks, geometric shapes associated with the self are identified more rapidly than comparable stimuli paired with other targets (e.g., friend, stranger). Replicating and extending work that challenges the putative automaticity of this effect, here we hypothesized that self-relevance facilitates stimulus processing only when task sets draw attention to previously formed shape–label associations in memory. The results of a shape-classification task confirmed this prediction. Compared to shapes associated with a friend, those paired with the self were classified more rapidly when participants were required to report who the stimulus denoted (i.e., self or friend). In contrast, self-relevance failed to facilitate performance when participants judged either what the shape was (i.e., triangle or square, diamond or circle) or where it was located on the screen (i.e., above or below fixation). These findings further elucidate the conditions under which self-relevance does—and does not—influence stimulus processing.

A commonly reported finding in the psychological literature is that self-relevance facilitates stimulus processing. Compared to material associated with other people (e.g., friend, mother, celebrity), items linked with the self are easier to notice, evaluate, and remember (e.g., Bargh & Pratto, 1986; Keyes & Brady, 2010; Kuiper & Rogers, 1979; Shapiro, Caldwell, & Sorensen, 1997; Symons & Johnson, 1997). Remedying a troublesome stimulus confound that undermined previous work on this topic (i.e., higher familiarity of own face/name vs. other face/name), recent research has demonstrated that the benefits of self-relevance extend even to inconsequential stimuli; notably, geometric shapes (Humphreys & Sui, 2016; Sui & Humphreys, 2017). After pairing shapes with various person-related labels (circle = you, triangle = friend, square = stranger), participants’ perceptual-matching judgments (i.e., do shape–label stimulus pairs match the previously learned associations?) were fastest for stimuli associated with the self (vs. best friend or stranger), a stimulus-prioritization effect that is claimed to reflect an obligatory facet of social-cognitive functioning (Janczyk, Humphreys, & Sui, 2019; Sui, He, & Humphreys, 2012; Sui, Liu, Mevorach, & Humphreys, 2013; Sui, Liu, Wang, & Han, 2009; Sui, Sun, Peng, & Humphreys, 2014). But is this necessarily the case?

There are at least a couple of reasons for questioning the alleged automaticity of self-prioritization during stimulus processing. First, a problematic aspect of the standard shape–label matching paradigm is that, throughout the task, participants must monitor the self-relevance of the stimuli (i.e., self-relevance is made salient through the experimental instructions), otherwise perceptual matching cannot be performed successfully (Sui et al., 2012, 2013, 2014). Indeed, when this explicit task requirement is removed, self-prioritization is eliminated (Dalmaso, Castelli, & Galfano, in press; Siebold, Weaver, Donk, & van Zoest, 2015; Stein, Siebold, & Zoest, 2016; Wade & Vickery, 2018). For example, in a rapid oculomotor search paradigm, Siebold et al. (2015) reported no facilitation in eye movements to lines previously associated with the self (vs. stranger). Similarly, using breaking continuous flash suppression (b-CFS) to examine the ease with which items (i.e., Gabors) access consciousness during a stimulus-localization task, Stein et al. (2016) observed no effect of self-relevance on the time taken for Gabors to overcome interocular suppression. Interestingly, however, contrasting Stein et al. (2016), Macrae, Visokomogilski, Golubickis, Cunningham, and Sahraie (2017) demonstrated enhanced access to visual awareness for self-relevant (vs. other-relevant) shapes when participants performed a stimulus-identification task. What this latter finding suggests is that self-prioritization is contingent upon task instructions (e.g., stimulus localization vs. stimulus identification) that draw attention to the self-relevance of target-object associations in memory.

Second, albeit in a different (though related) experimental context (i.e., object ownership), Falbén et al. (2019) reported that self-relevance only facilitated performance when semantic task sets (e.g., reporting the ownership or identity of pencils/pens) directed attention to previously formed target–object associations. When emphasis switched instead to a perceptual appraisal of the stimuli (i.e., reporting the orientation of pencils/pens), self-prioritization failed to emerge. Likewise, when required to report which of two objects initially appeared on the computer screen (i.e., temporal order judgment task)—a mug owned-by-self or a mug owned-by-the-experimenter—Constable, Welsh, Huffman, and Pratt (2019) revealed that participants were biased toward reporting that self-owned (vs. experimenter-owned) items were presented first. This effect was abolished, however, when the requested judgment was orthogonal to the dimension of interest (i.e., ownership), such that participants were asked to report whether a mug appeared to the left or right of fixation. Collectively these studies suggest that, during stimulus processing, self-prioritization is sensitive to the salience of self-object associations in memory.

Of course, ownership paradigms differ in several important respects from the shape–label matching tasks that have dominated work on this topic (Humphreys & Sui, 2016; Sui & Gu, 2017; Sui & Humphreys, 2015, 2017), but perhaps most significantly in terms of the status of the stimuli on which judgments are rendered (Constable et al., 2019; Falbén et al., 2019; Golubickis, Falbén, Cunningham, & Macrae, 2018; Sui et al., 2012, 2013, 2014; Truong, Roberts, & Todd, 2017). Whereas in perceptual-matching tasks geometric shapes serve as proxies for various social targets (e.g., self is a triangle, friend is a square); in ownership tasks, in contrast, everyday objects are linked with the self and others through association (e.g., self owns pens, friend owns pencils). This difference in the manner in which stimuli acquire self-relevance (i.e., self-surrogate vs. self-associate) may have important implications for the course and products of self-referential processing, hence the automaticity of self-prioritization (Humphreys & Sui, 2016; Sui & Humphreys, 2017). Accordingly, extending previous research (Constable et al., 2019; Falbén et al., 2019), here we considered whether task sets moderate self-prioritization when participants evaluate stimuli (i.e., geometric shapes) that denote the self and a friend (Sui et al., 2012).

Even when judging geometric shapes that designate the self (and others), we suspect that stimulus prioritization only occurs when the cognitive system is configured in particular ways. That is, like other seemingly automatic processes, self-prioritization is dependent on higher-order factors—including intentions, goals, and task sets—that shape stimulus processing and response generation (Kiefer, 2007; Memelink & Hommel, 2013; Moors & De Houwer, 2006; Naccache, Blandin, & Dehaene, 2002). In particular, whereas self-relevance should facilitate performance when task sets yield access to shape–target associations in memory (Janczyk et al., 2019; Sui et al., 2012, 2013, 2014); absent these specific processing conditions, self-prioritization is unlikely to emerge (Constable et al., 2019; Dalmaso et al., in press; Falbén et al., 2019; Siebold et al., 2015; Stein et al., 2016). Functioning in this way, self-prioritization is consistent with Hommel’s (1998, 2004, 2018) Theory of Event Coding (TEC). According to this account, processing episodes generate event files whereby elements of the experience are associated and stored in memory (e.g., self-is-a-square). Crucially, re-experiencing any of these stimuli (e.g., a square) at a later date can trigger partial or complete retrieval of the event file (e.g., the self-square association), thereby impacting task performance to varying degrees. In this way, the TEC provides a useful framework for understanding the generation of self-prioritization effects during stimulus processing (Hommel, 2004, 2018).

In a shape-classification task, here we considered how task sets impact self-prioritization (Constable et al., 2019; Falbén et al., 2019). Participants first associated geometric shapes (e.g., square, triangle) with either the self or a best friend (Sui et al., 2012). Next, to explore the automaticity of self-prioritization, they completed three blocks of trials in a computer-based shape-classification task, with each block requiring a different judgment to be undertaken on the stimuli. Specifically, participants reported: (i) to whom the shapes referred (‘who’ task); (ii) the identity of the shapes (‘what’ task); and (iii) whether the shapes appeared above or below the fixation cross on the screen (‘where’ task). In so doing, by probing different aspects of the stimuli (i.e., social status vs. physical status vs. perceptual status), the current experiment comprised a conceptual replication and extension of Falbén et al. (2019), in that the targets of judgment on this occasion were geometric shapes that served as proxies for the self and best friend (cf. self-owned vs. friend-owned objects). We expected self-prioritization to be moderated by the extent to which task sets access target–shape associations in memory during stimulus processing (Hommel, 2004; Memelink & Hommel, 2013).

## Method

### Participants and design

Eighty undergraduates (14 males, *M*_age_ = 20.67, *SD* = 3.06) took part in the research.^1^ All participants had normal or corrected-to-normal visual acuity. Five participants (1 male) were excluded from the statistical analysis for failing to follow the experimental instructions. Informed consent was obtained from participants prior to the commencement of the experiment and the protocol was reviewed and approved by the Ethics Committee at the School of Psychology, University of Aberdeen. The experiment had a 3 (Judgment: who vs. what vs. where) × 2 (Target: self vs. friend) × 2 (Shapes: triangle & square vs. diamond & circle) mixed design with repeated measures on the first two factors.

### Stimulus materials and procedure

Participants arrived at the laboratory individually, were greeted by an experimenter, seated in front of a desktop computer, and told they would be performing a shape-classification task. Following Sui et al. (2012), the experiment had two phases. The first phase comprised a learning task in which participants were required to associate geometric shapes with the self and a best friend. Participants were randomly assigned to one of the shape conditions. For half of the participants, the shapes were a triangle and a square; for the others they were a diamond and a circle. Two categories of shapes were used to provide a between-participants replication of the experiment. The shapes were not presented at this stage and shape–target associations and shape–target order were counterbalanced across the sample during the learning task.

Next, participants were informed they would be performing a shape-classification task. The task comprised three blocks of trials, with a different stimulus-related judgment required in each block. In one block (i.e., ‘who’ judgment), participants reported whether the shape presented on the screen represented them or their friend. In another block (i.e., ‘what ’judgment), whether the shape was a triangle or a square (diamond or circle). In a final block (i.e., ‘where’ judgment), whether the shape was displayed above or below the fixation cross on the screen. In all blocks, half of the items appeared above and half below the fixation cross and the order of the judgment tasks was counterbalanced across the sample. Before the start of each block, the experimenter explained that participants would be presented with a series of shapes and their task was simply to categorize each shape (via a button press), as quickly and accurately as possible, according to the specific judgment task. Responses were given using two buttons on the keyboard (i.e., N & M). Key-response mappings were counterbalanced across participants and across judgment tasks.

Each trial began with the presentation of a central fixation cross for 500 ms, followed by the image of a shape for 100 ms. After each shape was presented, the screen turned blank and participants had 1100 ms in which to respond (Sui et al., 2012). Following each response, the fixation cross reappeared and the next trial commenced. The images of the shapes were 214 × 214 pixels in size. Participants initially performed 8 practice trials for each type of judgment, followed by a block of 196 experimental trials in which stimuli occurred equally often in a random order. In total, there were 588 trials, with 196 trials in each block (i.e., judgment task), and 98 trials in each condition (i.e., 98 self-trials & 98-friend-trials). On completion of the task, participants were debriefed, thanked, and dismissed.

## Results and discussion

Responses faster than 200 ms were excluded from the analysis, eliminating less than 1% of the overall number of trials (see Table 1 for treatment means). A multilevel model was used to examine the response time (RT) and accuracy data. Analyses were conducted with the R package ‘lmer4’ (Pinheiro, Bates, DebRoy, Sarkar, & R Core Team, 2015), with participants as a crossed random effect (Judd, Westfall, & Kenny, 2012). Analysis of the RTs yielded main effects of Judgment (*b* = 0.376, *SE *= 0.621, *t  *= 60.49, *p* < 0.001) and Target (*b *= − 1.519, *SE *= 0.504, *t *= − 3.01, *p* = 0.003), and a significant Judgment × Target interaction (*b* = − 1.635, *SE* = 0.619, *t *= − 2.64, *p* = 0.008). Further analysis of the interaction revealed that, for ‘who’ judgments, RTs were faster for shapes that denoted the self than shapes that denoted a friend (*b* = − 3.465, *SE* = 0.906, *t *= − 3.83, *p* < 0.001). No such self-prioritization effect emerged on either ‘what’ (*b* = − 0.515, *SE* = 0.866, *t *= − 0.59, *p* = 0.552) or ‘where’ (*b* = − 0.311, *SE* = 0.717, *t *= − 0.43, *p* = 0.665) judgments.^2^

**Table 1:** Mean reaction time (RT) and accuracy (%) as a function of Judgment and Target

Judgment	Target	Mean RT (ms)	Accuracy (%)
Who	Self	408 (121)	89 (31)
	Friend	413 (121)	90 (30)
What	Self	407 (120)	92 (28)
	Friend	409 (120)	93 (26)
Where	Self	341(97)	97 (18)
	Friend	341 (95)	96 (19)

Standard deviations appear within parentheses

A multilevel logistic regression analysis on the accuracy of responses yielded main effects of Judgment (*b *= − 0.573, *SE* = 0.025, *z* = − 22.90, *p *< 0.001) and Target (*b *= − 0.41, *SE* = 0.020, *z* = − 2.02, *p *= 0.044). The Judgment X Target interaction, however, was not significant (*b *= − 0.013, *SE* = 0.025, *z* = 0.53, *p *= 0.594).

These results confirm that, across different pairs of shapes, self-prioritization is dependent on the task set that is operating during stimulus processing (Constable et al., 2019; Dalmaso et al., in press; Falbén et al., 2019; Siebold et al., 2015; Stein et al., 2016). Whereas self-prioritization was observed when judgments pertained to the target (i.e., ‘who’ judgment) with whom geometric shapes had previously been associated, no such effect emerged when they probed either the identity (i.e., ‘what’ judgment) or location (i.e., ‘where’ judgment) of the shapes. Moreover, the comparable RTs in the ‘who’ and ‘what’ (vs. ‘where’) tasks reveal that self-prioritization was not a function of task difficulty—stimulus prioritization only occurred when self-relevance was an explicit component of the task set (Siebold et al., 2015; Stein et al., 2016).

## Discussion

An extensive literature has demonstrated the benefits of self-relevance on stimulus processing (Humphreys & Sui, 2016; Sui & Humphreys, 2015). Using a perceptual-matching task, researchers have shown that participants make faster and less errant responses to stimuli related to the self than other persons. Critically, this effect has been replicated on numerous occasions, with self-prioritization extending to a wide range of stimuli, including: geometric shapes (Desebrock, Sui, & Spence, 2018; Enock, Sui, Hewstone, & Humphreys, 2018; Golubickis et al., 2017; Schäfer, Frings, & Wentura, 2016; Schäfer, Wentura, & Frings, 2017; Schäfer, Wesslein, Spence, Wenura, & Frings, 2016; Sui et al., 2012, 2013, 2014), badges of sports teams (Moradi, Sui, Hewstone, & Humphreys, 2015), objects (Schäfer, Wentura, & Frings, 2015), computer-generated avatars (Mattan, Quinn, Apperly, Sui, & Rothshtein, 2015), Gabor patches (Macrae, Visokomogilski, Golubickis, & Sahraie, 2018; Stein et al., 2016), lines (Siebold et al., 2015), and faces (Payne, Tsakiris, & Maister, 2017). In addition, self-prioritization has been reported across various sensory modalities (Frings & Wentura, 2014; Schäfer et al., 2015, 2016b).

Notwithstanding multiple demonstrations of self-prioritization during stimulus processing, lingering questions remain regarding the status of this effect. Based largely on studies using perceptual-matching tasks, conventional wisdom asserts that the mind is intricately tuned to personally meaningful material, such that self-relevance automatically enhances stimulus processing via bottom-up attentional capture (Humphreys & Sui, 2016; Sui & Humphreys, 2015; Sui & Rotshtein, 2019). Beyond shape–label matching tasks, however, the advantages of self-relevance appear to be far from obligatory. Across a range of paradigms and measures (e.g., oculomotor search, b-CFS, temporal order judgments, object categorization), self-relevance has been shown to exert little influence on task performance (e.g., Constable et al., 2019; Dalmaso et al., in press; Falbén et al., 2019; Siebold et al., 2015; Stein et al., 2016; Wade & Vickery, 2018), prompting Siebold et al. (2015, p. 1014) to conclude that “…the occurrence of the self-prioritization effect is specific to the requirements of the matching paradigm.” Echoing these sentiments, here we demonstrated the conditional automaticity of self-prioritization during a shape-classification task.

Replicating prior research, self-prioritization was contingent upon the task set (i.e., ‘who’ vs. ‘what’ vs. ‘where’) that was operating during stimulus evaluation (Constable et al., 2019; Dalmaso et al., in press; Falbén et al., 2019).^3^ Importantly, however, the current results diverged from Falbén et al. (2019) in an interesting way. Using an object-ownership task, Falbén et al. (2019) revealed that self-relevance facilitated performance when task sets probed either the ownership or physical identity of the stimuli (i.e., ‘who’ and ‘what’ tasks). When emphasis switched instead to a perceptual analysis of the objects (i.e., ‘where’ task), self-relevance had no influence on performance, thereby implying that self-prioritization requires the operation of semantic task sets. Here, in contrast, self-prioritization failed to materialize under a comparable semantic processing objective (i.e., ‘what’ is the shape?), emerging instead only when self-relevance was an overt aspect of the requested judgment (i.e., ‘who’ is the shape?). This difference highlights the task-related dependency of self-prioritization. Given the pivotal (and natural) role that proprietorship plays in everyday life (Beggan, 1992; Belk, 1988; 2014; James, 1890), ownership likely forges potent self-object connections in memory that can be accessed via multiple semantic pathways (Hommel, 2004). In contrast, the methodologically expedient—though somewhat artificial—activity of associating shapes with social targets is unlikely to create associations that can be reactivated in this way (Siebold et al., 2015; Stein et al., 2016), hence task sets must emphasize self-relevance to elicit stimulus prioritization. Thus, depending on the task context and the judgmental requirements, whether stimuli are self-surrogates or self-associates is an important determinant of prioritized processing.

Together with related research, the current findings underscore the malleability of self-prioritization (Constable et al., 2019; Dalmaso et al., in press; Falbén et al., 2019; Siebold et al., 2015; Stein et al., 2016; Wade & Vickery, 2018). Far from depicting a mandatory effect, the benefits of self-relevance appear sensitive to how stimuli are encountered, encoded, and evaluated (Bargh, 1989; Hommel, 2004, 2018; Moors & De Houwer, 2006). Of particular importance is the manner in which self-relevance is established. This, of course, would be expected if one assumes that self-referential processing guides behavior in a flexible way (e.g., Baars, 1988; Baumeister, 1998; Conway & Pleydell-Pearce, 2000; Heatherton, 2011; Neisser, 1988). If every potentially self-relevant stimulus was prioritized during processing, social-cognitive functioning would rapidly grind to halt. For example, absent the objective of driving into town, one’s car has no immediate applicability (i.e., goal-relevance). It would therefore be somewhat cumbrous if the mind was to prioritize this currently insignificant stimulus based only on its possible self-relevance. Under attentional control, self-relevance can guide cognition in a strategic, adaptive manner (Hommel, 2004, 2018).

The contextual flexibility that daily functioning demands may be provided by an intentional weighting mechanism that, prior to task performance (e.g., ‘who’ vs. ‘where’ tasks), increases the output gain on task-relevant features of the event file (i.e., target-object associations) in memory. In other words, depending on the task at hand, the same event file may be cognitively coded (i.e., weighted) in quite different ways, thereby producing quite different effects (see Memelink & Hommel, 2013). What is needed, therefore, are studies that tap the prioritization (or otherwise) of stimuli in task contexts that capture life outside the laboratory. Specifically, research must go beyond the explicit appraisal of inconsequential (though self-relevant) shapes to consider exactly when, how, and for whom different types of material are prioritized during stimulus processing (Golubickis et al., in press). In so doing, work of this kind will explicate the conditions under which self-relevance does—and indeed does not—facilitate thinking and doing.

## Footnotes

1 Sample size was based on Falbén et al. (2019).

2 To explore the possibility that task order influenced self-prioritization, we conducted an additional analysis of the RTs in which this repeated-measures factor was added to the multilevel model. The only additional effects to emerge in this analysis were a main effect of Order (*b* = − 6.420, *SE* = 0.636, *t *= − 10.10, *p* < 0.001) and a significant Order × Judgment interaction (*b* = − 6.889, *SE* = 1.083, *t *= − 6.36, *p* < 0.001). Further inspection of the interaction revealed an effect of Order on ‘who’ judgments (*b* = − 20.779, *SE* = 8.335, *t *= − 2.49, *p* = 0.015), such that responses (to both self and friend) were fastest (*M* = 393, *SD* = 115) when this task was performed third (vs. second  [*M* = 419, *SD* = 122] or first [*M* = 430, *SD* = 120]). Thus, task order did not impact self-prioritization.

3 The absence of a self-prioritization effect when judging the location of the stimuli could due to a floor effect in RTs (but see Falbén et al., 2019). Of greater theoretical interest, however, was the failure of the two semantic task sets to elicit comparable effects, despite RTs that were equivalent.
